# In-situ hydrothermal synthesis of CNT decorated by nano ZnS/CuO for simultaneous removal of acid food dyes from binary water samples

**DOI:** 10.1038/s41598-022-16676-4

**Published:** 2022-07-20

**Authors:** Ebrahim Sharifpour, Payam Arabkhani, Fatemeh Sadegh, Ali Mousavizadeh, Arash Asfaram

**Affiliations:** 1 Social Determinants of Health Research Center, Yasuj University of Medical Sciences, Yasuj, Iran; 2 Department of Chemistry, North Tehran Branch, Islamic Azad University, Tehran, Iran; 3 Department of Chemistry, Faculty of Sciences, University of Sistan of Baluchestan, Zahedan, Iran; 4 Medicinal Plants Research Center, Yasuj University of Medical Sciences, Yasuj, Iran

**Keywords:** Environmental sciences, Environmental social sciences, Materials science, Mathematics and computing, Nanoscience and technology

## Abstract

The zinc sulfide/copper oxide–carbon nanotube nanocomposite (ZnS/CuO-CNT) was fabricated by using an in-situ hydrothermal synthesis method and was used for simultaneous ultrasound-assisted adsorptive removal of a binary mixture of ponceau 4R (P4R) and tartrazine (TA) acid food dyes from contaminated water. The as-synthesized ZnS/CuO-CNT was described by FESEM, XRD, FTIR, BET, and zeta potential analysis. The results included nested network morphology, high purity with the crystalline structure, oxygen-containing functional groups, mesoporous/micropores texture with cumulate interspace, specific surface area of 106.54 m^2^ g^-1^, and zero-point charge (pH_zpc_) of 5.3. In adsorption experiments, the simultaneous effect of main independent variables, including solution pH, adsorbent dosage, concentration of each dye, temperature, and sonication time on the removal efficiency of dyes was studied systematically using the central composite design (CCD) method based on response surface methodology (RSM). Also, the second-order multivariate equation was presented to determine the relationship between the removal efficiencies of P4R and AT dyes and six independent effective variables. The high correlation coefficient (R^2^ ≥ 0.99), significant *p*-value (*P* < 0.0001), and non-significant lack-of-fit (*P* > 0.05) showed the high accuracy, and validity of the proposed model to predict the removal efficiency of P4R and TA acid food dyes. The experimental removal efficiency for P4R and TA dyes was found to be 98.45 ± 2.54, and 99.21 ± 2.23, respectively. Also, the Langmuir maximum adsorption capacity for P4R and TA dyes was determined to be 190.1 mg g^-1^ and 183.5 mg g^-1^, respectively. Finally, the adsorbent's reusability was tested for six periods and could be reused repeatedly without significant reduction in adsorption performance.

## Introduction

In general, one of the methods for determining the quality of food items is the color and flavor of the food. Changes in these two parameters, according to science, can influence the nutritional and therapeutic qualities of the product in many situations. Dyes are chemicals used in the food business to improve the quality of the goods produced. They offer the required foundation for a competitive market for food items^1,2^. As a result, dyes might be considered an important category of food additives. According to the source of production, these compounds (dyes) are classified into two categories: natural and artificial. Synthetic dyes do not have similar natural dyes. They are in fact, compounds that are synthesized using chemical methods and have an unlimited range of applications and are used in the dyeing, pharmaceutical, microbiological, food, and other industries. The instability and high sensitivity of natural dyes to light, temperature, pH, etc., cause many changes in this category of dyes while preparing and storing food, so artificial dyes have received more attention in the food industry^3^. On the other hand, researchers have demonstrated that these chemicals are detrimental to human health^4^. Having continuous usage or absorption via the skin, many synthetic dyes, particularly those with azo functional groups and aromatic rings in their structure, can cause chromosomal damage^5^, allergies^6^, asthma^7^, thyroid tumors^8^, and respiratory illnesses^9^. Research has also shown that daily consumption of foods containing food additives has also been found to lower children's IQ by five points, according to research^10^. Ponceau 4R (P4R) and Tartrazine (TA) are two anionic synthetic food dyes that are always used together to form an orange dye^11^. P4R and TA have been widely used to make groceries more attractive and tasty because of their exceptional benefits such as low cost, high solubility in water, down microbiological pollution, and high consistency^12^. Nonetheless, P4R and TA belong to the azo dyes (–N = N–) with an aromatic and carcinogenic structure that can lead to side effects such as allergies, cancer, and neurological behaviors if consumed in excess^13–15^. In scientific references, different removal methods such as membrane methods^16^, electrochemical and sonochemical^17^, ion exchange resins^18^, coagulation^19^, flocculation, ^20^ nanofiltration^21^, and reverse osmosis^22^ have been used to remove synthetic dyes. However, these methods have limitations such as high cost, sludge formation, or by-products limiting their use^23^. With the advancement of science and technology, the adsorption process has recently been regarded for the treatment of different organic compounds as well as heavy metal ions in contaminated water owing to its excellent qualities such as low raw material costs, ease of design, safety, and efficiency^24^. Researchers have studied the removal of dyes with different adsorbents^25,26^. Among the adsorbents used, carbon nanotubes (CNTs) are widely used to remove various contaminants due to their high surface area to volume and high adsorption capacity^27^. Also, different types of metal oxides/sulfides nanoparticles have been combined with carbon nanotubes to remove a variety of contaminants from wastewaters^28,29^. Recently, applications of ultrasound for wastewater treatment have been conducted by many researchers in ultrasound-assisted adsorption^30^ and can be used as a very effective technique as an accelerating agent in increasing the adsorption capacity. The increased adsorption capacity in the presence of ultrasound is defined due to the effect of ultrasound in reducing the agglomeration of nanoparticles, increasing kinetics, and enhancing the affinity between adsorbent and adsorbate^31,32^. Nowadays, many statistical experimental design methods are applied for the optimization of process parameters. In the conventional one-variable-at-a-time statistical method, the optimization of a multivariable system follows one factor at a time and does not represent the combined effect, and is not able to study the interactions and complexities between independent variables^33^. On the other hand, the conventional optimization technique requires more experiments to determine the optimum conditions and takes prolonged time^34^. However, the experimental design method based on the central composite design (CCD) method along with response surface methodology (RSM) is a mathematical and statistical technique that is able to optimize and model the complex relationships between variables and system responses with a limited number of experiments^35^.

In this study, the carbon nanotubes were decorated by nano zinc sulfide/copper oxide (ZnS/CuO) through the in-situ hydrothermal method to fabricate the zinc sulfide/copper oxide–carbon nanotube nanocomposite (ZnS/CuO-CNT) and was used as an efficient adsorbent for simultaneous ultrasound-assisted removal of dye mixture P4R and TA from contaminated water. The structural characteristics of adsorbent was studied in detail by field emission scanning electron microscopy (FESEM), X-ray diffraction (XRD), Fourier transform infrared spectroscopy (FTIR), Brunauer–Emmett–Teller (BET), and zeta potential (ZP) analysis. The adsorptive removal process was performed on a laboratory scale based and optimized by the central composite design (CCD) method based on response surface methodology (RSM). The effects of independent variables such as solution pH, adsorbent dosage, initial concentration of each dye, sonication time, and temperature at different levels were evaluated. Finally, the adsorption isotherms, kinetics, thermodynamics, reusability, and efficiency in real samples were investigated.

## Experimental section

### Materials and instruments

Carbon nanotubes (CNTs) with the length of 5–15 µm and outside diameter of < 15 nm were procured from Nanoshel, USA. Nitric acid (HNO_3_, 70%), copper (II) sulfate pentahydrate (CuSO_4_.5H_2_O), hexamethylenetetramine (HMTA, C_6_H_12_N_4_), zinc nitrate hexahydrate (Zn(NO_3_)_2_⋅6H_2_O), thioacetamide (CH_3_CSNH_2_), cetyltrimethylammonium bromide (CTAB, CH_3_(CH_2_)_15_ N(Br)(CH_3_)_3_), absolute ethanol (CH_3_CH_2_OH), hydrochloric acid solution (HCl, 37%), and sodium hydroxide (NaOH, pellets) were all purchased from Sigma-Aldrich without any impurities. The purity and crystal structure of the samples were identified by X-ray diffraction (XRD) analysis (Philips X'Pert MPD, Netherlands, Cu Kα). The surface functional groups of the samples were determined by Fourier-transform infrared (FTIR) spectroscopy (Thermo Nicolet, Avatar 360, USA). The surface morphology of the samples were investigated by the field emission scanning electron microscope (FESEM; Zeiss, SIGMA VP-500, Germany). The specific surface area, total pore volume, and mean pore diameter were measured by nitrogen adsorption–desorption isotherm with multi-point Brunauer − Emmett − Teller (BET) method (Bel Japan Inc., Belsorp mini-II). An ultrasonic bath with the heating system (Sonorex, Bandelin, Berlin, Germany) at the frequency of 40 kHz was used for the ultrasound-assisted adsorption. The concentration of dyes was measured by a ultraviolet–visible (UV–Vis) spectrophotometer (HACH, DR 5000, USA). The zeta potential (ZP) of the composite was measured at 25.0 ± 0.5 °C by using a Zetasizer 2000 (Malvern Instruments Ltd., Worcestershire, UK).

### Preparation

#### Functionalization of carbon nanotubes

The chemical oxidation route was carried out by HNO_3_ acid treatment as follows^36^: 100 mL HNO_3_ (15 mol L^-1^) was prepared and added into a round bottom flask. Then, 100 mg of pristine carbon nanotubes were dispersed into the acid solution by ultrasonication for 30 min and then refluxed for 5 h in an oil bath at 120 ºC. After centrifugation at 5000 rpm for 10 min, the HNO_3_-treated CNTs were recovered and washed with ultrapure water until the pH of the CNT solution neared neutral. Finally, the functionalized carbon nanotubes (CNTs) were dried in the oven at 120 ºC overnight.

#### Synthesis of CuO-CNT nanocomposite

The CuO-CNT nanocomposite was synthesized by the in-situ hydrothermal method as follows: The 0.7 g of dried CNTs were suspended in 50 mL of ultrapure water by sonicating for 30 min to obtain the homogeneous dispersion of carbon nanotubes. Then, the resulting homogeneous dispersion of the CNTs was mixed with 50 mL of CuSO_4_.5H_2_O solution under constant stirring. Then, 5 mL of the aqueous solution of hexamethylenetetramine (HMTA) was added with continuous stirring. The homogenous solution was then transferred to a 100 mL stainless autoclave lined with Teflon and heated to 170 °C for 12 h. After that, the autoclave was allowed to cool at room temperature and black precipitates were collected and washed with ethanol and ultrapure water several times. Finally, the CuO-CNT nanocomposite was obtained successfully and was kept for the subsequent synthesis.

#### Synthesis of ZnS/CuO-CNT nanocomposite

The ZnS/CuO-CNT nanocomposite was obtained by the in-situ hydrothermal method as follows^37^: At first, certain amounts of Zn(NO_3_)_2_⋅6H_2_O (2 mmol), thioacetamide (2 mmol), and CTAB (2 mmol) were dissolved in 50 mL of ultrapure under magnetic stirring. Then, the obtained above solution was added to CuO-CNT dispersion solution and was stirred for 30 min. The solution was then transferred to a 100 mL stainless autoclave lined with Teflon and heated to 170 °C for 12 h. Finally, the collected precipitate was calcined under an argon atmosphere at 400 °C for 1 h, and ZnS/CuO-CNT nanocomposite was synthesized successfully.

### Food dyes adsorption experiments

**Figure 1:**
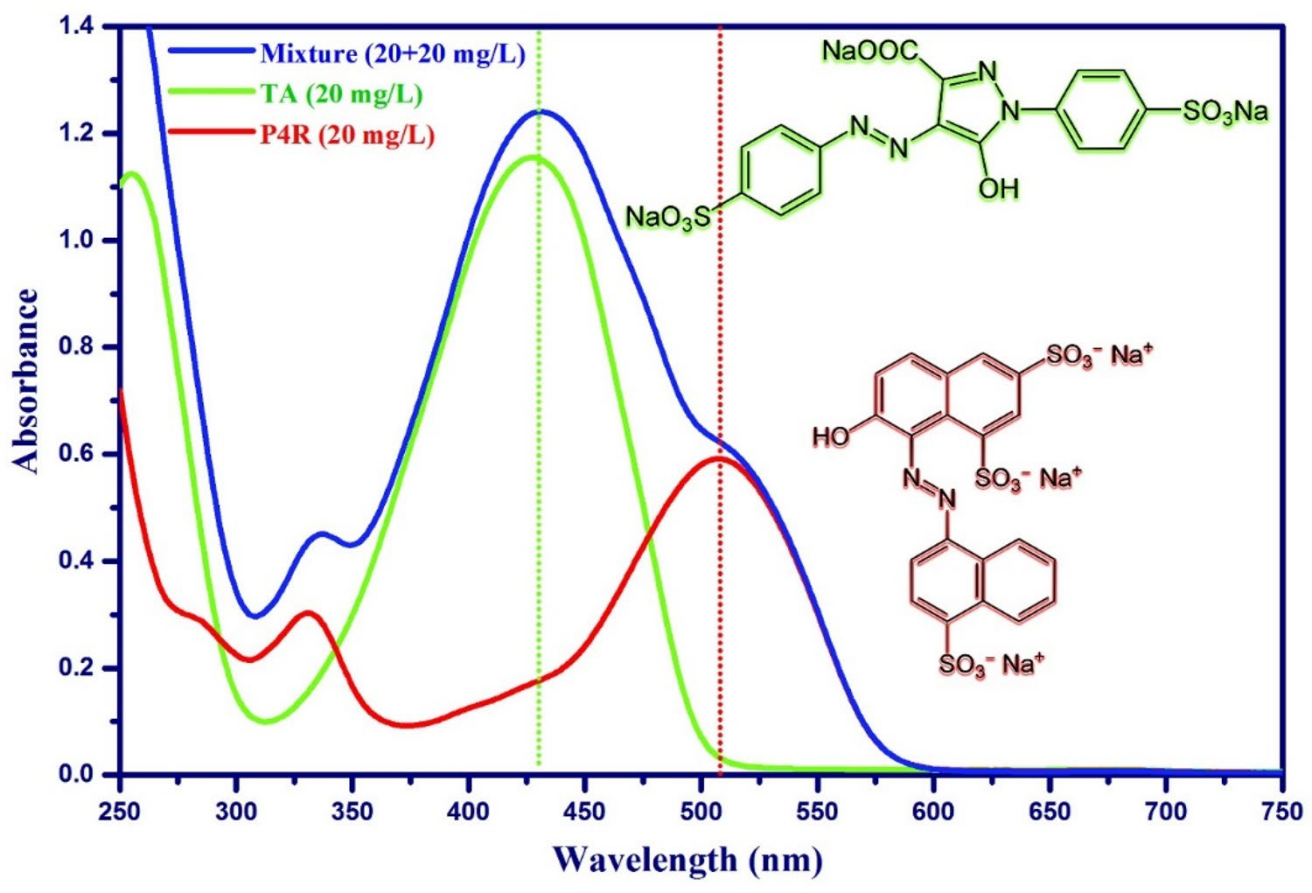
The representative UV–V is spectra (zero-order derivative) for TA, P4R, and mixture of dyes with related chemical structures.

**Figure 2:**
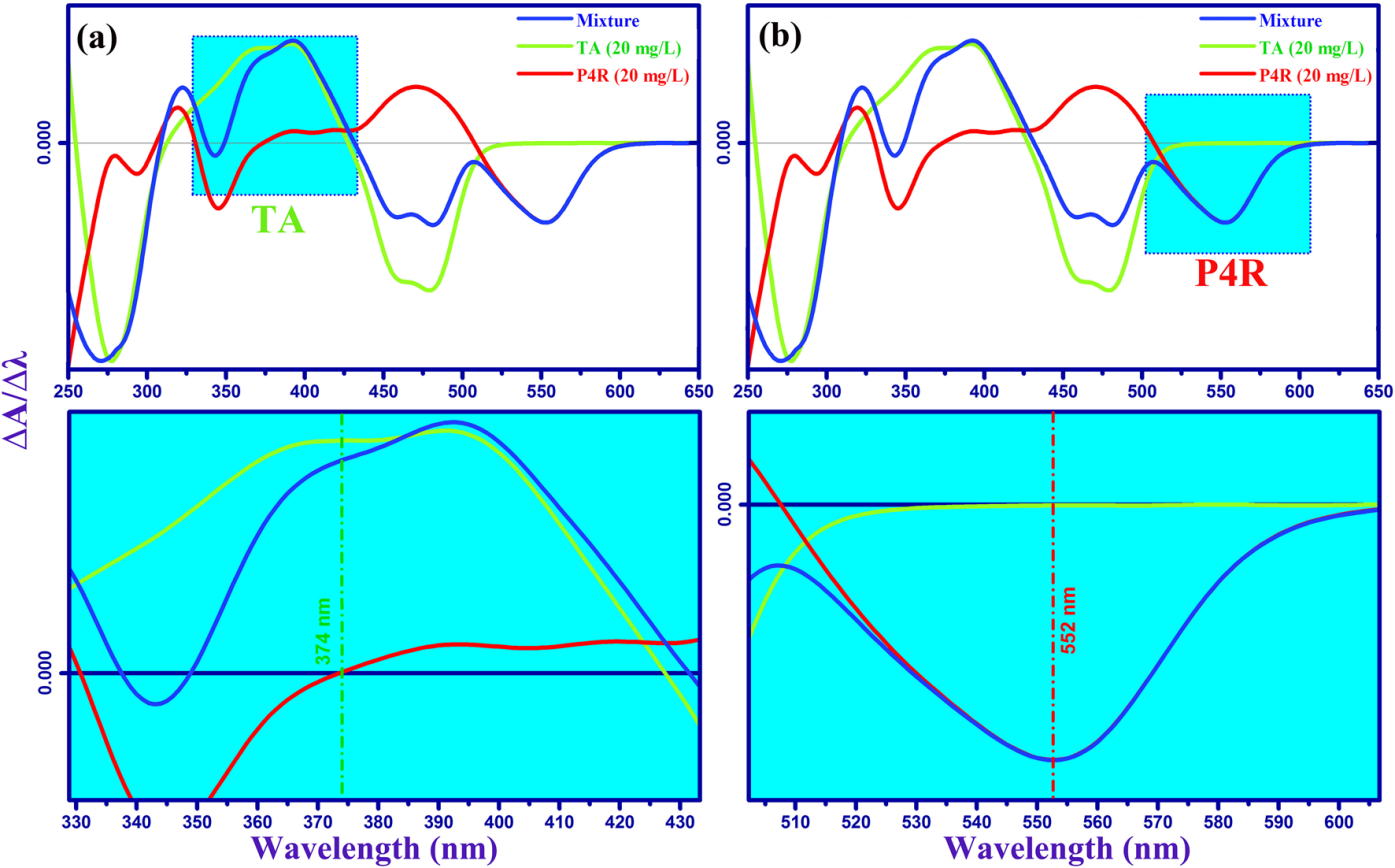
The representative first order derivative spectrum of TA (**a**), P4R (**b**), and mixture of dyes in aqueous solution.

**Figure 3:**
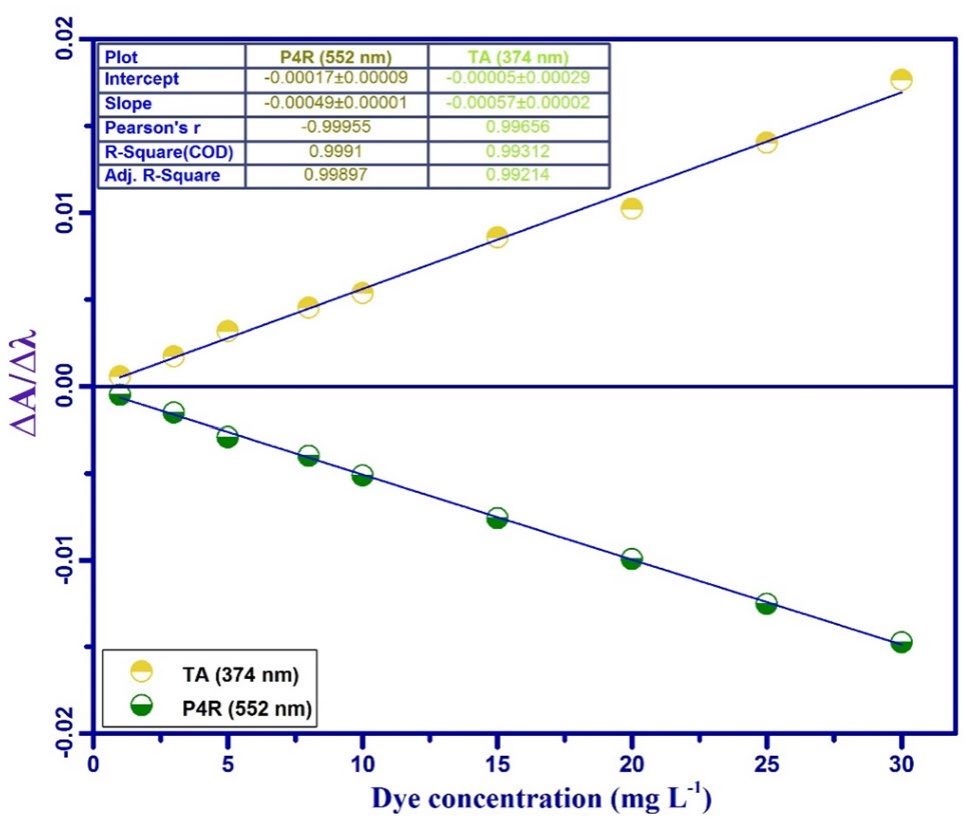
Calibration curve of TA (374 nm) and P4R (552 nm) dyes.

Batch sorption experiments were carried out in the presence of ultrasound power (40 kHz / 100 W) in an ultrasonic cleaning bath. The adsorption tests were carried out in the 100 mL cylindrical jacketed glass vessel. Using a UV–Vis spectrophotometer, the final concentration of dyes in treated solutions was determined after the adsorption process was completed. The Design-Expert software (Version 12.0.1, Stat-Ease Inc., Minneapolis, MN) was used in the experimental design and statistical analysis. The STATISTICA software (Version 10.0) was used to generate response surface plots. The experiments were performed based on the CCD matrix (Table 1) and in random order. In all experiments, 50 ml of a mixture of dyes P4R and TA was used at room temperature. For quantitative measurements through spectroscopy, the maximum wavelength of species adsorption in solution must be determined. Therefore, the zero-order absorption spectra of the solution of P4R and TA dyes, each with a concentration of 20 mg L^-1^ and a binary mixture of dyes in the range of 250 to 750 nm, were determined (Fig. 1). Because the absorption spectra of P4R and TA dyes overlapped and displayed interference between their zero-order spectra, direct absorbance measurements could not be used to quantify their amounts simultaneously. First-order derivative spectrophotometry was created to tackle this challenge and determine the concentration of P4R and TA in binary mixes (Fig. 2). The use of zero-order derivative spectroscopy to separate overlapping signals and decrease the influence of spectral interposition caused by the attendance of another ingredient in a sample could be beneficial^38^. By detecting the absorbance signal at the first-order derivative wavelength, the residual concentrations of P4R and TA dyes in the binary mixture were monitored aided the simultaneous removal of dyes. The method of zero-crossing was used to choose two wavelengths for two dyes. The proper wavelength of P4R is 552 nm, for which the first-order derivative of TA is zero. According to the first-order derivative method, the correct wavelength of TA is 374 nm (Fig. 2). As a result, the calibration curve for P4R and TA dyes was determined using the dye's first-order derivative spectrum at specified wavelengths (Fig. 3). The absorbance of the solution was then determined by UV–Vis spectroscopy at the specified wavelengths and the removal percentage was calculated by the below equation:

1 $$R\% = \frac{{C_{0} - C_{t} }}{{C_{0} }} \times 100\%$$

**Table 1:** Experimental design matrix using RSM-CCD and average responses for adsorptive removal of TA and P4R dyes by ZnS/CuO-CNT.

Independent variables		Range and levels (coded)						
Coded	Factors	−α	Low		Middle	High		+ α
X_1_	pH	2.0	4.0		6.0	8.0		10
X_2_	Sonication time (min)	2.0	5.5		9.0	12.5		16.0
X_3_	Adsorbent mass (mg)	5.0	7.5		10.0	12.5		15
X_4_	Temperature (°C)	5.0	15		25	35		45
X_5_	TA concentration (mg L^-1^)	10	20		30	40		50
X_6_	P4R concentration (mg L^-1^)	10	20		30	40		50

Run order	Adsorption variables						Responses (R%)	
	X_1_	X_2_	X_3_	X_4_	X_5_	X_6_	R% TA	R% P4R
1	6.0	9.00	10.0	25	10	30	99.87	96.38
2	10	9.00	10.0	25	30	30	37.65	31.03
3	8.0	5.50	7.5	35	20	20	59.87	49.87
4	2.0	9.00	10.0	25	30	30	99.76	96.27
5	6.0	9.00	10.0	25	30	50	85.98	48.18
6	8.0	12.5	7.5	35	20	40	69.87	33.25
7	8.0	5.50	7.5	15	40	20	26.45	54.34
8	6.0	9.00	5.0	25	30	30	40.87	32.38
9	6.0	9.00	10.0	25	30	30	66.36	66.31
10	4.0	12.5	12.5	35	40	20	95.67	99.33
11	4.0	5.50	7.5	35	40	20	80.87	98.87
12	6.0	9.00	15.0	25	30	30	88.98	81.16
13	8.0	12.5	12.5	35	20	20	70.65	65.56
14	6.0	9.00	10.0	25	30	30	68.45	62.65
15	4.0	12.5	7.5	15	20	40	94.54	80.65
16	8.0	5.50	12.5	35	20	40	50.45	26.76
17	6.0	16.0	10.0	25	30	30	78.98	68.38
18	4.0	12.5	7.5	35	40	40	66.00	70.56
19	6.0	9.00	10.0	5	30	30	57.76	63.37
20	6.0	9.00	10.0	25	30	30	68.56	63.73
21	6.0	9.00	10.0	25	30	30	68.89	64.67
22	8.0	12.5	12.5	15	40	20	45.98	76.76
23	4.0	5.50	7.5	15	20	20	90.87	93.76
24	6.0	9.00	10.0	25	30	10	88.98	96.40
25	4.0	5.50	12.5	15	20	40	87.87	68.87
26	4.0	12.5	12.5	15	20	20	98.67	95.67
27	6.0	9.00	10.0	25	50	30	25.54	86.38
28	6.0	2.00	10.0	25	30	30	27.65	18.16
29	8.0	12.5	7.5	15	40	40	35.65	20.98
30	4.0	5.50	12.5	35	40	40	71.98	89.87
31	6.0	9.00	10.0	45	30	30	85.54	78.38
32	8.0	5.50	12.5	15	40	40	50.98	54.87

In this equation, C_0_ is the concentration of dye in the initial solution and C_t_ is the concentration of dye in the final solution.

The accuracy of the first-order derivative method was determined by evaluating the recovery percentage by considering a binary mix of P4R and TA dyes with different concentrations. Table 2 shows a summary of the results. The recoveries and errors for the designation of the P4R and TA dyes in the binary mix using the first-order derivative spectroscopic technique were 96–103 percent and − 3.17 and + 3.62 percent, respectively, as shown in Table 2. Based on recovery perusal, the first-order derivative spectroscopic technique can correctly identify the amounts of P4R and TA dyes in the binary mixture^39^.

**Table 2:** Results of percentage recovery and error values of TA and P4R standard dye mixture by the first-order derivative spectrophotometric method.

Mixture (mg L^-1^)	TA			P4R		
	Founded (mg L^-1^)	Recovery (%)	Error (%)	Founded (mg L^-1^)	Recovery (%)	Error (%)
16 (TA) + 9 (P4R)	16.12	100.75	0.75	8.921	99.12	− 0.88
28 (TA) + 1 (P4R)	27.45	98.04	− 1.96	0.994	99.40	− 0.60
1 (TA) + 35 (P4R)	0.981	98.10	− 1.90	35.67	101.91	1.91
4 (TA) + 12 (P4R)	4.12	103.00	3.00	11.69	97.39	− 2.61
12 (TA) + 4 (P4R)	11.84	98.67	− 1.33	4.10	102.50	2.50
8 (TA) + 25 (P4R)	7.88	98.48	− 1.53	25.61	102.44	2.44
16 (TA) + 12 (P4R)	16.31	101.94	1.94	11.69	97.38	− 2.63
20 (TA) + 20 (P4R)	20.61	103.05	3.05	19.88	99.38	− 0.62
30 (TA) + 5 (P4R)	31.08	103.60	3.60	4.91	98.20	− 1.80
6 (TA) + 35 (P4R)	5.81	96.83	− 3.17	35.88	102.50	2.50
25 (TA) + 15 (P4R)	24.76	99.04	− 0.96	15.54	103.62	3.62
2 (TA) + 40 (P4R)	1.95	97.65	− 2.35	39.43	98.58	− 1.43
35 (TA) + 18 (P4R)	34.39	98.26	− 1.74	17.83	99.06	− 0.94
40 (TA) + 2 (P4R)	38.87	97.18	− 2.83	2.05	102.60	2.60
20 (TA) + 30 (P4R)	20.31	101.55	1.55	30.83	102.77	2.77
	${\mathbf{\overline{X} = }}{99}{.94} \pm {0}{.60; }{\mathbf{SD = }}{ 2}{.33; }{\mathbf{RSD = }}{ 2}{.34\% }$			${\mathbf{\overline{X} = }}{100}{.5} \pm {0}{.57; }{\mathbf{SD = }}{ 2}{.20; }{\mathbf{RSD = }}{ 2}{.19\% }$		

${{\mathbf{\overline{X}}}}$: Mean ± **SE**: Standard Error; **SD**: Standard Deviation; **RSD**: Relative Standard Deviation

### Experiment design

To evaluate the performance of the ZnS/CuO-CNT adsorbent, a discontinuous system and experimental design method were used. For this purpose, the CCD-RSM design was applied for five independent variables with five levels. The solution pH, adsorbent dosage, initial concentration of each dye, ultrasound time, and temperature were considered the independent variables and removal percentage was considered dependent variables. Table 1 shows the independent variables, their range and experimental levels. In general, in this study, the number of experiments was 32. The results of experimental work in different periods were used to prepare a predictable model. This equation is a polynomial regression model discussed in previous studies^40^.

## Results and discussions

### Characterization

**Figure 4:**
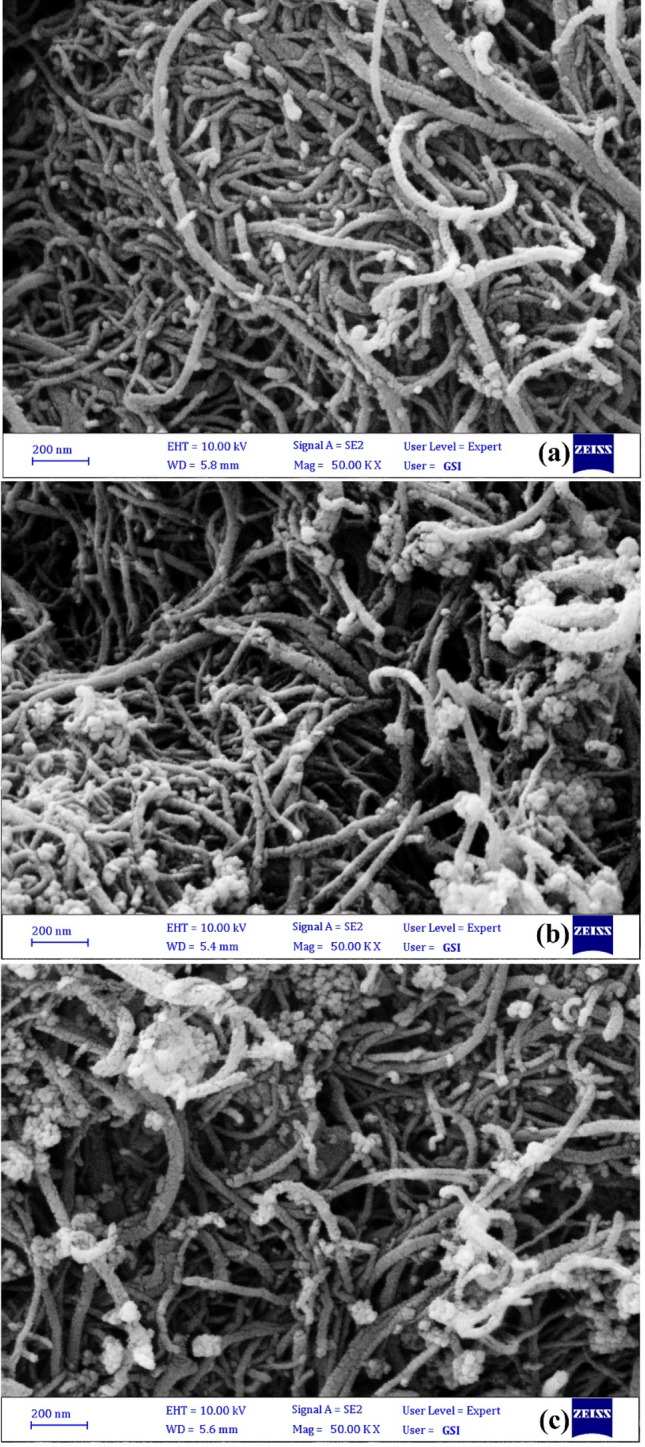
FESEM images of CNT (**a**), CuO-CNT (**b**), and ZnS/CuO-CNT (**c**).

The surface morphology of the samples was investigated by the FESEM images and are shown in Fig. 4. In the FESEM image of CNT (Fig. 4a), nanotubes are seen as filaments of micrometer length and nanometer thickness that are densely intertwined with an almost smooth surface. In the FESEM image of CuO-CNT (Fig. 4b), it was observed that the CuO nanoparticles with a near-spherical shape and average diameter of about 20–40 nm were formed in the CNTs network. Also about ZnS/CuO-CNT (Fig. 4c), the growth of almost spherical nanoparticles with a size of less than 50 nm in the between and surface of the nanotubes can still be seen, which has created a nested network morphology.

**Figure 5:**
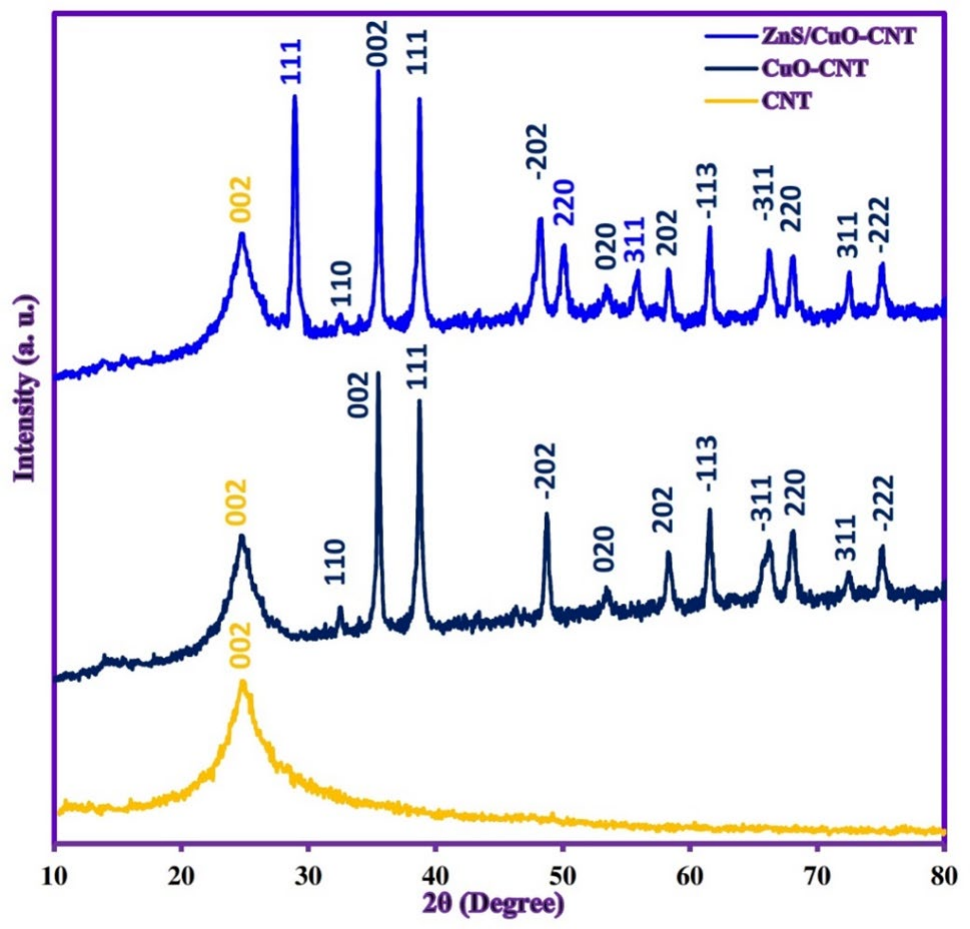
XRD patterns of CNT, CuO-CNT, and ZnS/CuO-CNT.

**Figure 6:**
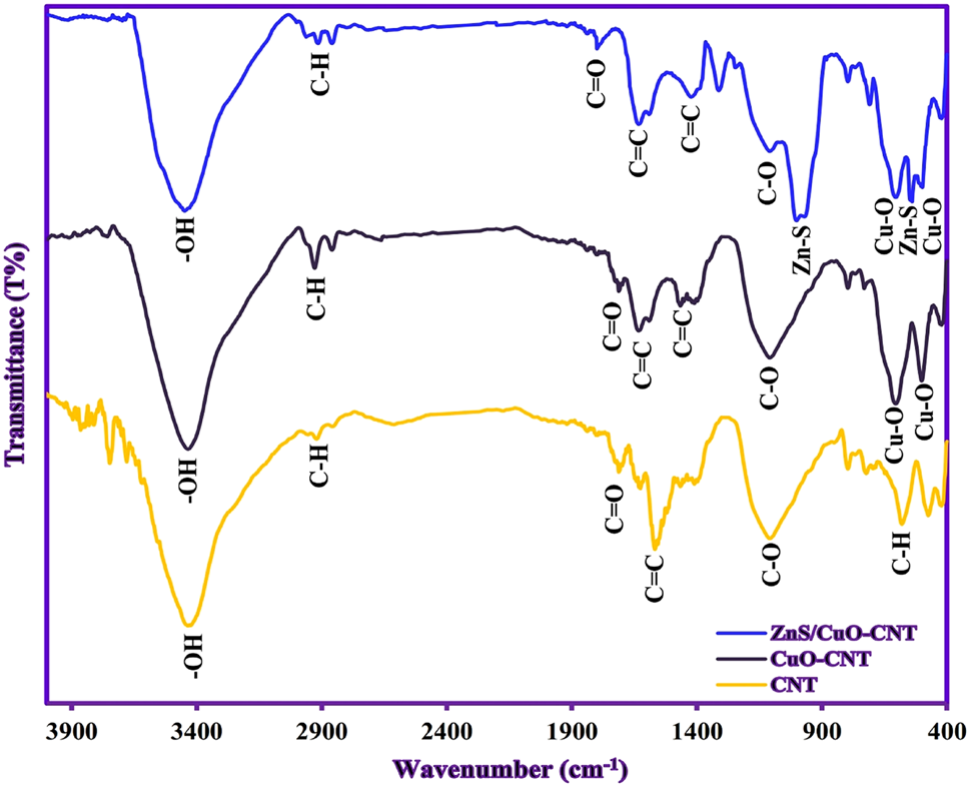
FTIR spectra of CNT, CuO-CNT, and ZnS/CuO-CNT.

**Figure 7:**
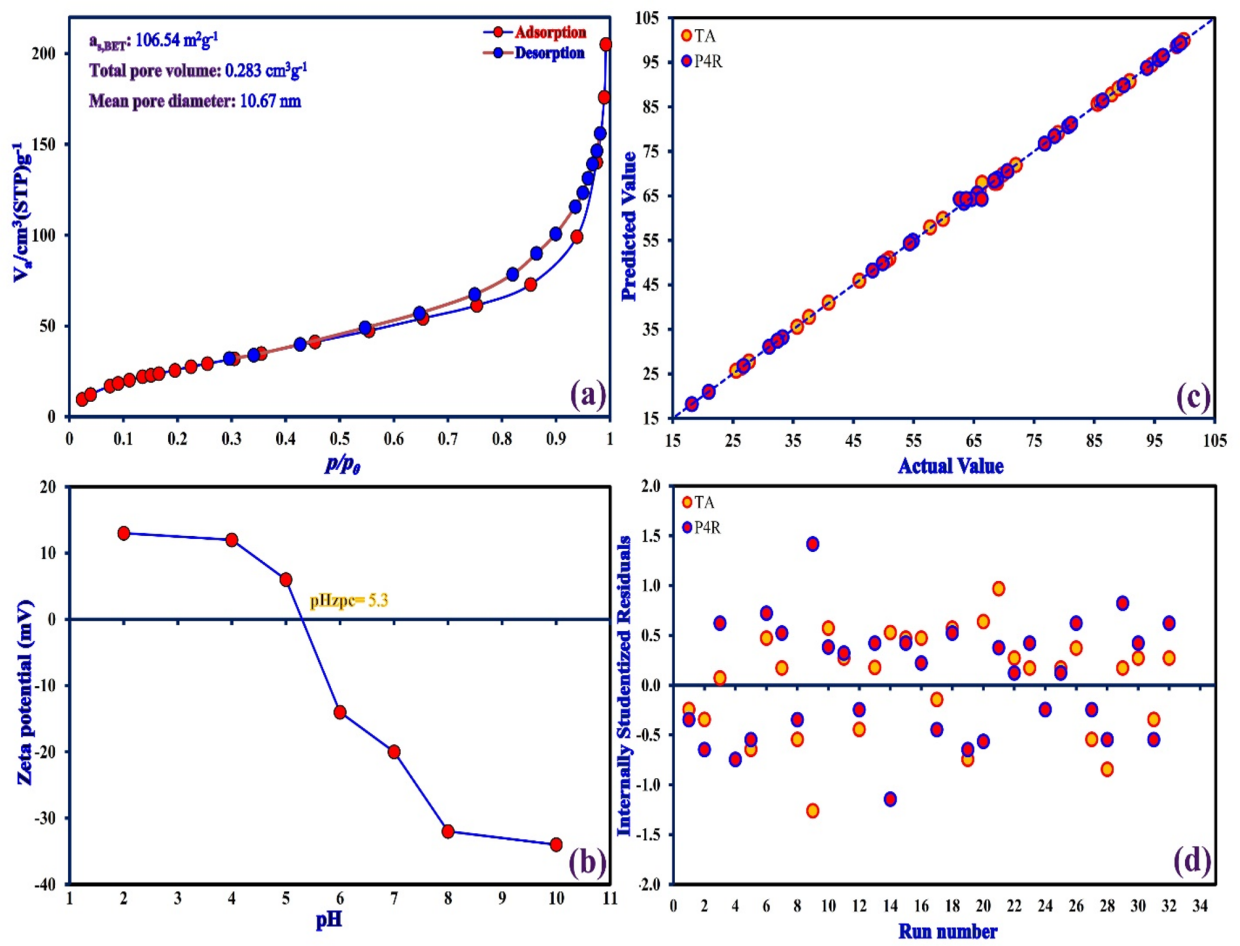
(**a**) Nitrogen adsorption/desorption isotherm for ZnS/CuO-CNT, (**b**) The zeta potential of ZnS/CuO-CNT as a function of pH, (**c**) The actual vs. predicted values plot, and (**d**) The residual vs. observation order plot for removal of TA and P4R dyes.

The crystal phases and purity of samples were analyzed by XRD patterns and are shown in Fig. 5. The XRD pattern of CNT (Fig. 5a) showed the main diffraction peak at about 2θ = 26.1°, attributed to the characteristic peak of the (002) crystal plane of CNTs^41^. In the XRD pattern of CuO-CNT (Fig. 5b), the new characteristic peaks have appeared along with CNT peaks which were assigned to (110), (002), (111), (− 202), (020), (202), (− 113), (− 311), (220), (311), and (− 222) crystal planes of monoclinic CuO (JCPDS no. 45–0937)^42^. The sharp diffraction peaks indicated that the CuO nanoparticles were grown with a high crystalline structure in the presence of nanotubes. The XRD pattern of ZnS/CuO-CNT (Fig. 5c) showed the appearance of new diffraction peaks compared to the XRD pattern of CuO-CNT, which attributed to the (111), (220), and (311) crystal planes of cubic ZnS phase (JCPDS no. 80–0020)^43^. Also, no additional diffraction peaks were seen indicating that the synthesis of ZnS/CuO-CNT was successful. The FTIR measurements were conducted to evaluate the surface functional groups of samples and the results are shown in Fig. 6. In the CNT spectrum (Fig. 6a), the bands at 610 cm^-1^, 1100 cm^−1^, 1565 cm^-1^, and 1640 cm^-1^ can be assigned to C–H (out of plane bending), C–O, C = C (aromatic rings), and C = O (–COOH) stretching vibrations, respectively^44^. In the FTIR spectrum of CuO-CNT (Fig. 6b), the presence of Cu–O absorption bands along with CNT bands was verified at wavenumber 480–580 cm^−1^^45^. In the FTIR spectrum of ZnS/CuO-CNT (Fig. 6c), the Zn–S stretching vibrations were assigned at 497 and 998 cm^−1^, which confirmed the successful formation of ZnS in presence of CuO-CNT composite^46^. Also, the absorption peaks related to absorbed water molecules around 1640 cm^-1^ were not shown in the figure due to the possibility of overlapping with C = O absorption peaks. The textural properties of the ZnS/CuO-CNT were studied by nitrogen adsorption–desorption isotherms (Fig. 7a) and defined as a type II isotherm with H3 hysteresis loop, which indicates the presence of both mesopores and micropores texture with cumulate interspaces in the sample^47^. The BET model also estimated the specific surface area, total pore volume, and mean pore diameter of ZnS/CuO-CNT, which were 106.54 m^2^ g^-1^, 0.283 cm^3^ g^-1^, and 10.67 nm, respectively.

The zeta potential of ZnS/CuO-CNT was determined as a function of pH, and the result are presented in Fig. 7b. It is observed that the zeta potential of ZnS/CuO-CNT in the range of pH 4.0 to 8.0 is more strongly dependent on pH and its value becomes from + 12 to − 32 mV as the solution becomes more alkaline. Also, the zero value of the zeta potential (isoelectric point) was obtained and the pH of the zero point charge (pH_zpc_) of the ZnS/CuO-CNT was 5.3.

### Modeling and optimization of adsorption

The modeling and optimization of adsorption process was performed by the CCD-RSM design, and adequacy of the model was tested through the analysis of variance (ANOVA). The ANOVA results based on the quadratic model are presented in Table 3.

**Table 3:** ANOVA of the regression model for the prediction of dyes removal yield.

Source of variation	DF	TA				P4R			
		SS	MS	F-value	*P*-value	SS	MS	F-value	*P*-value
Model	27	16,292	603.40	548.91	< 0.0001	18,460	683.7	376.7	< 0.0001
X_1_	1	1928.8	1928.8	1754.7	< 0.0001	2128	2128	1172	< 0.0001
X_2_	1	1317.4	1317.4	1198.4	< 0.0001	1261	1261	694.7	< 0.0001
X_3_	1	1157.3	1157.3	1052.8	< 0.0001	1190	1190	655.5	< 0.0001
X_4_	1	385.86	385.86	351.02	< 0.0001	112.7	112.7	62.06	0.0014
X_5_	1	2762.5	2762.5	2513.0	< 0.0001	50.0	50.0	27.55	0.0063
X_6_	1	4.5000	4.5000	4.0936	0.1131	1162	1162	640.4	< 0.0001
X_1_X_2_	1	7.7145	7.7145	7.0179	0.0570	15.74	15.74	8.672	0.0422
X_1_X_3_	1	1.1610	1.1610	1.0562	0.3622	193.3	193.3	106.5	0.0005
X_1_X_4_	1	457.01	457.01	415.74	< 0.0001	173.2	173.2	95.44	0.0006
X_1_X_5_	1	122.78	122.78	111.70	0.0005	107.6	107.6	59.29	0.0015
X_1_X_6_	1	154.32	154.32	140.38	0.0003	68.02	68.02	37.47	0.0036
X_2_X_3_	1	18.365	18.365	16.706	0.0150	0.424	0.4243	0.2338	0.6540
X_2_X_4_	1	25.883	25.883	23.545	0.0083	0.0770	0.0770	0.0424	0.8469
X_2_X_5_	1	62.608	62.608	56.954	0.0017	273.8	273.8	150.9	0.0003
X_2_X_6_	1	433.86	433.86	394.68	< 0.0001	298.6	298.6	164.5	0.0002
X_3_X_4_	1	35.551	35.551	32.341	0.0047	19.08	19.08	10.51	0.0316
X_3_X_5_	1	249.25	249.25	226.74	0.0001	368.2	368.2	202.8	0.0001
X_3_X_6_	1	454.01	454.01	413.05	< 0.0001	794.9	794.9	437.9	< 0.0001
X_4_X_5_	1	16.485	16.485	14.997	0.0180	61.27	61.27	33.75	0.0044
X_4_X_6_	1	194.81	194.81	177.22	0.0002	0.2426	0.2426	0.1336	0.7332
X_5_X_6_	1	3.0888	3.0888	2.8099	0.1690	0.3335	0.3335	0.1837	0.6903
X_1_^2^	1	1.5675	1.5675	1.4259	0.2984	0.6591	0.6591	0.3631	0.5793
X_2_^2^	1	380.25	380.25	345.91	< 0.0001	800.4	800.4	441	< 0.0001
X_3_^2^	1	14.784	14.784	13.449	0.0214	101.8	101.8	56.08	0.0017
X_4_^2^	1	27.280	27.280	24.817	0.0076	79.75	79.75	43.94	0.0027
X_5_^2^	1	46.764	46.764	42.541	0.0029	1338	1338	737.2	< 0.0001
X_6_^2^	1	705.87	705.87	642.13	< 0.0001	117.5	117.5	64.75	0.0013
Residual	4	4.3971	1.0993			7.26	1.815		
Lack of Fit	1	0.4162	0.4162	0.3136	0.6146	0.0425	0.0425	0.0177	0.903
Pure Error	3	3.9809	1.3250			7.218	2.406		
Corr. Total	31	16,296				18,470			

Model summary statistics									
	Mean (R%)	SD	C.V%	R^2^	Adj-R^2^	Pred-R^2^	Adequate Precision (AP)		
TA	68.32	1.048	1.5354	0.999	0.998	0.961	75.79		
P4R	66.68	1.347	2.020	0.999	0.997	0.996	64.36		

DF: Degrees of Freedom; SS: Sum of Squares; MS: Mean Square; SD: Standard Deviation; RSD: Relative Standard Deviation; C.V: Coefficient of variation; R^2^:R-squared; Adj-R^2^: Adjusted-R^2^; Pred-R^2^: Predicted R-squared.

The coefficient R^2^ represents the ratio of the total changes in the response predicted by the model, which represents the ratio of the sum of squares due to regression (SSR) to the sum of squares (SST). A large R^2^ and close to 1 are desirable, and a favorable agreement with the essential R^2^-Adj. The large R^2^ confirms the satisfactory conformity of the test data to the quadratic model^48^. F-value is an indicator to evaluate the significance of the model that the larger the numerical value, and the, more significant the model and *P*-value are less than 0.05 approved by the model^49^. The value of *P*-related factors greater than 0.1 do not have a significant effect on the model and by removing them, the model can be modified. As shown in Table 3, the fitting weakness test for the quadratic polynomial model fitted to the surface response data is not significant, indicating a successful fit of the data. Therefore, high correlation coefficient (R^2^ ≥ 0.99), significant *p*-value (*P* < 0.0001), and non-significant lack-of-fit (*P* > 0.05) indicate high accuracy, and validity of the proposed model to predict the efficiency of simultaneous removal of dyes mixture by ZnS/CuO-CNT from aqueous solutions.

Multi-nominal regression modeling between the response variable (removal %) and the corresponding coded values (X_1_, X_2_, X_3_, X_4_, X_5_, and X_6_) of five different variables, respectively (solution pH, ultrasound time, adsorbent dosage, temperature, initial concentration of each dye) was created and finally, the best-fitted model was obtained as follows:

2 $$\begin{aligned} R\%_{TA}^{{}} =& + 297.9 - 10.2X_{1} - 0.7X_{2} - 9.5X_{3} + 3.8X_{4} - 3.9X_{5} - 0.5X_{1} X_{4} + 0.2X_{1} X_{5} + 0.2X_{1} X_{6} \hfill \\& +{ }0.2X_{2} X_{3} + 0.04X_{2} X_{4} - 0.1X_{2} X_{5} + 0.3X_{2} X_{6} - 0.1X_{3} X_{4} + 0.2X_{3} X_{5} + 0.4X_{3} X_{6} \hfill \\&+ { }0.02X_{4} X_{5} - 0.03X_{4} X_{6} - 0.3X_{2}^{2} - 0.1X_{3}^{2} + 0.01X_{4}^{2} - 0.01X_{5}^{2} + 0.05X_{6}^{2} \hfill \\ \end{aligned}$$

3 $$\begin{aligned} R\%_{P4R}^{{}} = &+ 362.6 - 12.5X_{1}^{{}} + 7.9X_{2}^{{}} - 12.3X_{3}^{{}} + 0.6X_{4}^{{}} - 7.4X_{5}^{{}} - 8.7X_{6}^{{}} + 0.2X_{1}^{{}} X_{2}^{{}} + 0.7X_{1}^{{}} X_{3}^{{}} \hfill \\ &-{ }0.3X_{1}^{{}} X_{4}^{{}} + 0.2X_{1}^{{}} X_{5}^{{}} - 0.1X_{1}^{{}} X_{6}^{{}} - 0.1X_{2}^{{}} X_{5}^{{}} + 0.2X_{2}^{{}} X_{6}^{{}} - 0.04X_{3}^{{}} X_{4}^{{}} + 0.2X_{3}^{{}} X_{5}^{{}} \hfill \\ &+{ }0.5X_{3}^{{}} X_{6}^{{}} + 0.04X_{4}^{{}} X_{5}^{{}} - 0.4X_{2}^{2} - 0.3X_{3}^{2} + 0.02X_{4}^{2} + 0.07X_{5}^{2} + 0.02X_{6}^{2} \hfill \\ \end{aligned}$$

Negative coefficients imply a diminishing influence of factors on dye adsorption, whereas positive coefficients suggest an increasing effect of variables^50^.

Also, the actual values plotted against the predicted values derived from the CCD model of dyes removal efficiency are shown in Fig. 7c. As can be seen, the plot indicated a good agreement between the actual and predicted values for the removal efficiency of dyes, which indicated the efficiency and accuracy of the predicted model. In addition, the residuals need to be observed to ensure that the ANOVA assumptions are valid. The residual error analysis was studied by the residuals vs observation order plot as shown in Fig. 7d. It shows that the residuals in the plot fluctuate around the centerline and don't explicitly show a trend. Therefore, the randomization in the experiment was vindicated suggesting that the ANOVA assumptions are valid.

### Simultaneous effect of independent variables on dyes removal efficiency

**Figure 8:**
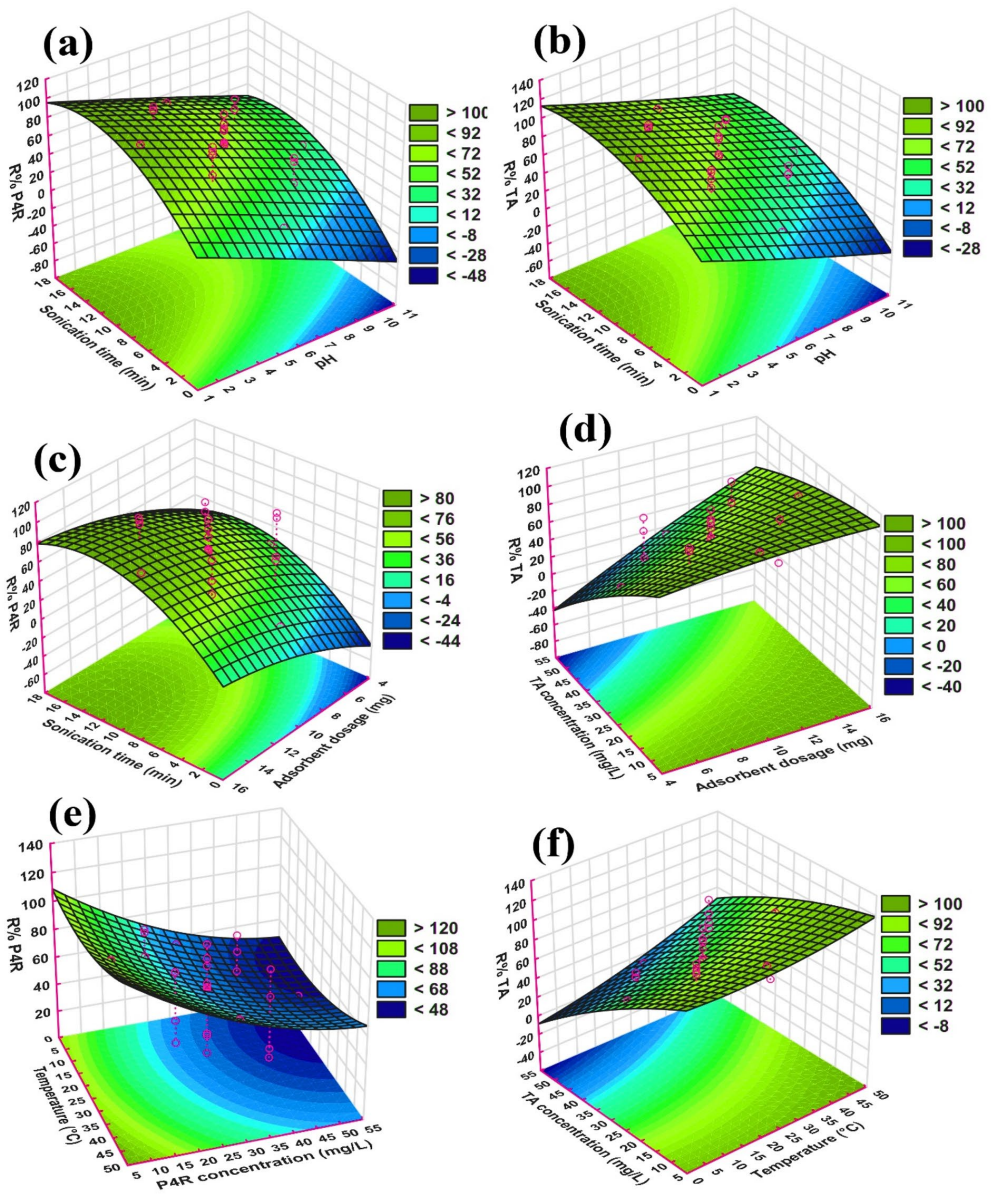
Three-dimensional response surface plots of independent variables on the adsorptive removal efficiency of TA and P4R dyes by ZnS/CuO-CNT: (**a**, **b**) pH-sonication time, (**c**, **d**) adsorbent dosage-sonication time/concentration, and (**e**, **f**) temperature-dye concentration.

The simultaneous effect of independent variables such as pH (2.0–10), adsorbent dosage (5–15 mg), initial concentration of each dye (10–50 mg L^-1^), sonication time (2–16 min), and the temperature (5–45 °C) on P4R/TA dyes removal efficiency was displayed by the three-dimensional response surface diagrams as shown in Fig. 8. As can be seen, three-dimensional response surface diagrams were performed as a function of two independent variables that change in laboratory ranges and the third variable was the removal efficiency of the dyes, which was reported as a removal percentage (R%). The simultaneous effects of sonication time and solution pH on R% of P4R and TA are shown in Fig. 8a,b, respectively. It was observed that the R% has increased at lower pH ranges as well as longer sonication times in both dyes. The P4R and TA dyes are anionic due to the presence of sulphonic groups in their structures. Also, in acidic conditions, the adsorbent surface has a positive charge due to its zeta potential and pH_zpc_. Therefore, the generated electrostatic attraction can play an effective role in the adsorption process and increase the removal efficiency in acidic conditions. In contrast, with the increase of pH and more became alkaline, the positively charged sites of the adsorbent get decreased and trends to create the negatively charged. This situation leads to electrostatic repulsion between the negatively charged surface of the adsorbent and anionic dye causing a decrease in adsorption of the dyes from the aqueous solution. Similar findings were also reported in other studies^51,52^. Further, ultrasonic waves contribute to faster mass transfer processes, increase the diffusion coefficient, and also increase the chemical reaction of particles in solution by increasing the effective collisions interparticle collisions^53,54^. Therefore, the removal efficiency was increased with increasing the sonication time. On the other hand, the equilibrium adsorption time was very fast in occurred in 12 min. Rapid adsorption is due to the surface bonding of the species to the adsorbent surface. It is believed that when the adsorption process is rapid, the speed limiting step is related to the transfer process in the liquid phase, such as penetration into the solution mass, penetration from the vicinity of the adsorb film to solid particles, or penetration into cavities filled with solution^55^. The simultaneous effects of sonication time and adsorbent dosage on R% of P4R is shown in Fig. 8c. As can be seen, with increasing sonication time and adsorbent dosage the removal efficiency of dye has increased until it has finally reached a state of equilibrium. The rate of rapid adsorption in the first minutes of the reaction can be interpreted as due to the presence of very high adsorption sites and the high concentration gradient of contaminant molecules. Gradually, however, the adsorption sites become saturated and the adsorption rate is controlled by the phenomenon of transfer from the outside to the inside of the adsorbent. Also, it is obvious that with increasing the amount of adsorbent, the number of available vacant active sites on the adsorbent increases compared to dye molecules and more adsorption sites remain unsaturated during the adsorption process causing the removal efficiency was increased^56^. In the adsorption process by ZnS/CuO-CNT, the simultaneous effect of adsorbent dosage and initial dye concentration on R%TA is illustrated in Fig. 8d. It has been shown that the R%TA increased with decreasing TA dye concentration and increasing the adsorbent dosage. In fact, in high dye concentrations, the vacant sites of the adsorbent are saturated and increase competition in adsorption between the dye molecules. Therefore, the lack of surfaces and active sites for adsorption at high concentrations causes less adsorption to be done and reduces the removal efficiency^57^. The simultaneous effect of temperature and initial dye concentration on R% of dyes are seen in Fig. 8e,f. The result indicates the highest removal efficiency took place in higher temperatures with lower dyes concentrations. It can be assumed that increasing the temperature increases the kinetic energy of the reacting particles and increases the probability of collisions between dye molecules and the adsorbent surface, as a result, the dye removal efficiency increases^58^. It can be assumed that increasing the temperature increases the kinetic energy of the reacting particles and increases the probability of collisions between dye molecules and the adsorbent surface, as a result, the dye removal efficiency increases, which in line with other previous reports^58,59^.

### Optimum conditions of the adsorption process

**Figure 9:**
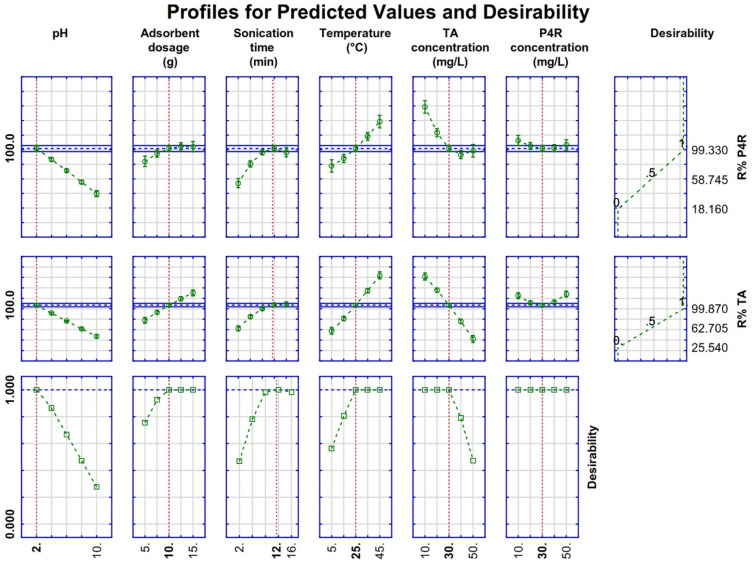
Profiles for predicted values and desirability function for the maximum R % of dyes. Dotted red lines indicate the optimization values.

The desirability function (DF) was used to optimize the values of the independent variables to get the high performance of the ZnS/CuO-CNT in P4R and TA dyes removal by applying a scale ranging from 0.0 (undesirable) to 1.0 (very desirable). The profiles for predicted values and desirability function for the maximum R% of dyes are shown in Fig. 9. The dotted red lines indicate the optimum values with a desirability function of 1.0, which were found at solution pH: 2.0, adsorbent dosage: 10 mg, sonication time: 12 min, dyes concentration: 30.0 mg L^-1^, and solution temperature: 25 °C. Under the optimum conditions, the predicted R% response for TA and P4R dyes was above 99.0% with a desirability function of 1.0. Also, the experimental R% for TA and P4R were found to be 99.21 ± 2.23, and 98.45 ± 2.54, respectively. Therefore, the predicted results were closely correlated with the experimental data obtained from desirability optimization analysis using CCD-RSM.

### Adsorption isotherm models

The adsorption isotherm is critical for describing how the contaminant interacts with the adsorbent and for explaining the adsorbent's adsorption capability. Langmuir, Freundlich, Temkin, and Dubinin-Radushkevich's models were used to analyze the adsorption isotherm. The coefficients of the equations and the isothermal parameters were obtained by calculating the slope and breadth of the regression line in the isotherm diagrams of the adsorption of each dye by the adsorbent from the origin. Table 4 shows the results of these computations as well as the coefficients for the isothermal equations of P4R and TA dyes. According to the results, the Langmuir isotherm is most consistent with the adsorption process for each of the P4R and TA dyes, with a correlation coefficient of 0.999, followed by the Temkin, Freundlich, and Dubinin-Radushkevich isotherms. In the Langmuir model, it is assumed that the adsorbed molecules have no interaction and that the surface adsorption is a monolayer. In the Langmuir relation, K_L_ (L mg^-1^) is the equilibrium constant of surface adsorption, C_e_ (mg L^-1^) is the equilibrium concentration of the adsorbent, q_e_ (mg g^-1^) is the adsorbent's equilibrium adsorption capacity, and q_m_ (mg g^-1^) is the adsorbent's maximum adsorption capacity. The Langmuir isotherm, by defining a dimensionless constant called the separation coefficient (R_L_), indicates whether the process of adsorption is desirable or undesirable. For desirable adsorption, the R_L_ value is between 0 and 1. Indicate undesirable surface adsorption, linear adsorption, and irreversible adsorption are all indicated by R_L_ values larger than one, and R_L_ = 1 and R_L_ = 0, respectively^60^. The value of the R_L_ coefficient for adsorption of P4R and TA dyes on ZnS/CuO-CNT were 0.002 and 0.003, respectively.

**Table 4:** Estimated equilibrium parameters of different isotherms for the adsorptive removal of TA and P4R dyes by ZnS/CuO-CNT.

Isotherm	Plot	Parameters	P4R	TA
Langmuir $\frac{{C_{e} }}{{q_{e} }} = \frac{1}{{Q_{m} k_{L} }} + \frac{{C_{e} }}{{Q_{m} }}$	*C*_*e*_*/q*_*e*_* vs. C*_*e*_	Q_m_ (mg g^-1^)	190.1	183.5
		K_L_ (L mg^-1^)	11.04	5.807
		R^2^	0.999	0.999
		R_L_ = 1/(1 + (K_L_ × C_0_))	0.002	0.003
		Chi-square (χ^2^)	2.1E-07	2.8E-7
Freundlich $lnq_{e} = lnK_{F} + \frac{1}{n}lnC_{e}$	*ln q*_*e*_* vs. ln C*_*e*_	1/n	0.238	0.220
		K_F_ (L mg^-1^)	8.516	8.122
		R^2^	0.849	0.965
		Chi-square (χ^2^)	0.015	0.003
Temkin $q_{e} = B_{1} ln K_{T} + B_{1} ln C_{e}$	*q*_*e*_* vs. ln C*_*e*_	B_1_	22.74	18.933
		K_T_(L mg^-1^)	1.000	1.000
		R^2^	0.965	0.944
		Chi-square (χ^2^)	145.2	206.3
Dubinin-Radushkevich $ln q_{e} = lnQ_{s} - k\varepsilon^{2}$	*ln q*_*e*_* vs. ε*^*2*^	Q_s_ (mg g^-1^)	171.1	139.7
		β × 10^–8^	− 1.112	− 0.61
		E (kJ mol^-1^)	6.704	9.054
		R^2^	0.943	0.825
		Chi-square (χ^2^)	0.029	0.086

Freundlich isotherm is an isotherm that describes non-ideal and reversible surface adsorption. This experimental model is used for multilayer surface adsorption as well as for heterogeneous surfaces. The K_F_ (L mg^-1^) constant in the Freundlich relation is the absorption isotherm constant, which is related to the adsorption capacity, and n is the Freundlich isotherm constant, which is related to the adsorption severity. If 1/n = 0 the adsorption process is irreversible, if 0 < 1/n < 1 the adsorption process is desirable and if 1/n > 1 the adsorption process is undesirable^60^. As can be seen in the table, the value of 1/n for P4R and TA dyes were equal to 0.238 and 0.220, respectively, and indicates the desirability of the adsorption process.

The Temkin isotherm has a factor that includes absorbed-adsorbent interactions. This model considers that the adsorption heat of all molecules on the surface decreases with increasing surface coverage. The quantity of K_T_ (mg L^-1^) in the Temkin isotherm is the constant of the and B_1_ (mol J^-1^) is the quantity related to the heat of surface adsorption. The Dubinin-Radushkevich isotherm is an experimental model. In the Dubinin-Radushkovich model, if the amount of adsorption energy (Kj mol^-1^) is less than 8, the adsorption is of the physical type due to weak van der Waals forces, If it is in the range of 8–16, the adsorption follows the ion exchange mechanism, and If E is between 20 and 40, chemical adsorption has taken place^61,62^. The E value calculated in the Dubinin-Radushkovich isotherm for TA dye was 9.054 kJ mol^-1^ and shows the presence of ion exchange mechanism (E > 8 kJ mol^-1^). For the P4R dye, the E value was 6.704 kJ mol^-1^, suggesting that P4R dye has been adsorbed mainly via physisorption (E < 8 kJ mol^-1^).

The nonlinear Chi-square (χ^2^) test was used to assess the fitness of isotherm models in addition to the correlation coefficient of determination (R^2^). If the data from a model and the experimental data are comparable, χ^2^ is a small number; if they are significantly different, χ^2^ is a large number^63^. As can be seen, the Langmuir model exhibited lower χ^2^ values than other isotherms, which lead to a better fit of the calculated data from the Langmuir model and experimental results.

### Adsorption kinetic studies

The various kinetic models were used to study the adsorption kinetics (Table 5). According to the First-order-kinetic model, infiltration occurs inside a boundary layer. This model is based on the adsorbent capacity, which states that variations in the quantity of adsorption over time are proportionate to the number of vacant places on the adsorbent level. The pseudo-second-order-kinetic model is based on solid-phase adsorption and indicates that chemical adsorption is the primary control mechanism in the adsorption function. It also places that the chemical adsorption steps slow down the adsorption process. Weber-Morris intraparticle diffusion model is used to study the mechanism of the adsorption kinetics. The quantitative adsorption process may be controlled by one or more steps, including external penetration or penetration into the film, penetration into the pores, penetration into the level, and adsorption on the surface of the pores, or a combination thereof. In a solution that is stirred rapidly, mass transfer through diffusion can be expressed by an apparent diffusion coefficient calculated from experimental data^64,65^. Elovich model is also one of the most widely used models and unlike previous models that are compatible with physical adsorption, it shows the chemical interaction between adsorbate and adsorbent^66^. According to the given models, the factors calculated from each model are reported in Table 5. In these equations, q_e_ (mg g^-1^) and q_t_ (mg g^-1^) are the adsorption capacities at equilibrium time and time of the t, respectively. k_1_ (min^-1^), k_2_ (g mg^-1^ min^-1^), and h (mg g^-1^ min^-1^) are the first-order velocity constant, second-order velocity constant, and initial sorption rate, respectively. K_diff_ (mg g^-1^ min^0.5^) is the penetration rate constant, and C (g mg^-1^) is a constant that gives an idea of the thickness of the boundary layer. Also, α and β are the indicators of the Elovich model^67^. The results show that the adsorption process follows the Pseudo-second-order-kinetic model based on the R^2^ value of P4R (0.996) and TA (0.996) dyes. Also, the amount of equilibrium capacity calculated (q_e, calc_) from the Pseudo-second-order-kinetic model is more consistent with the equilibrium capacity obtained from experiments (q_e, exp_). Therefore, a better fit of the results with second-order kinetics model indicated that the chemical adsorption was the primary control mechanism in the adsorption process.

**Table 5:** Kinetic parameters and correlation coefficient for different adsorption kinetic models for TA and P4R dyes adsorption.

Model	Plot	Parameters	P4R	TA
First-order- kinetic $\ln (q_{e} - q_{t} ) = \ln q_{e} - k_{1} t$	*ln (q*_*e*_* − q*_*t*_*) vs. t*	k_1_ (min^-1^)	0.408	0.378
		q_e (calc)_ (mg g^-1^)	124.5	107.7
		R^2^	0.899	0.947
		Chi-square (χ^2^)	0.029	0.012
Pseudo-second-order-kinetic $\frac{t}{{q_{t} }} = \frac{1}{{k_{2} q_{e}^{2} }} + \frac{t}{{q_{e} }}$	*t/q*_*t*_* vs. t*	k_2_ (min^-1^)	0.004	0.005
		q_e (calc)_ (mg g^-1^)	166.8	162.5
		R^2^	0.996	0.996
		Chi-square (χ^2^)	1.8E-6	1.9E-6
		h (mg g^-1^ min^-1^)	121.4	136.6
Intraparticle diffusion $q_{t} = k_{diff} t^{1/2} + C$	*q*_*t*_* vs. t*^*1/2*^	K_diff,1_ (mg g^-1^ min^-1/2^)	48.03	53.44
		C_1_ (mg g^-1^)	30.56	19.85
		R_1_^2^	0.983	0.932
		K_diff,2_ (mg g^-1^ min^-1/2^)	28.11	26.18
		C_2_ (mg g^-1^)	62.83	67.46
		R_2_^2^	0.994	0.999
		K_diff,3_ (mg g^-1^ min^-1/2^)	10.23	7.858
		C_3_ (mg g^-1^)	114.6	123.1
		R_3_^2^	0.794	0.871
		Chi-square (χ^2^)	46.83	26.42
Elovich $q_{t} = \frac{1}{\beta }\ln (t) + \frac{1}{\beta }\ln (\alpha \beta )$	*q*_*t*_* vs. ln t*	β (g mg^-1^)	0.032	0.035
		α (mg g^-1^ min^-1^)	348.2	477.0
		R^2^	0.993	0.997
		Chi-square (χ^2^)	7.093	2.140
Experimental data		q_e (exp)_ (mg g^-1^)	147.2	146.2

**Figure 10:**
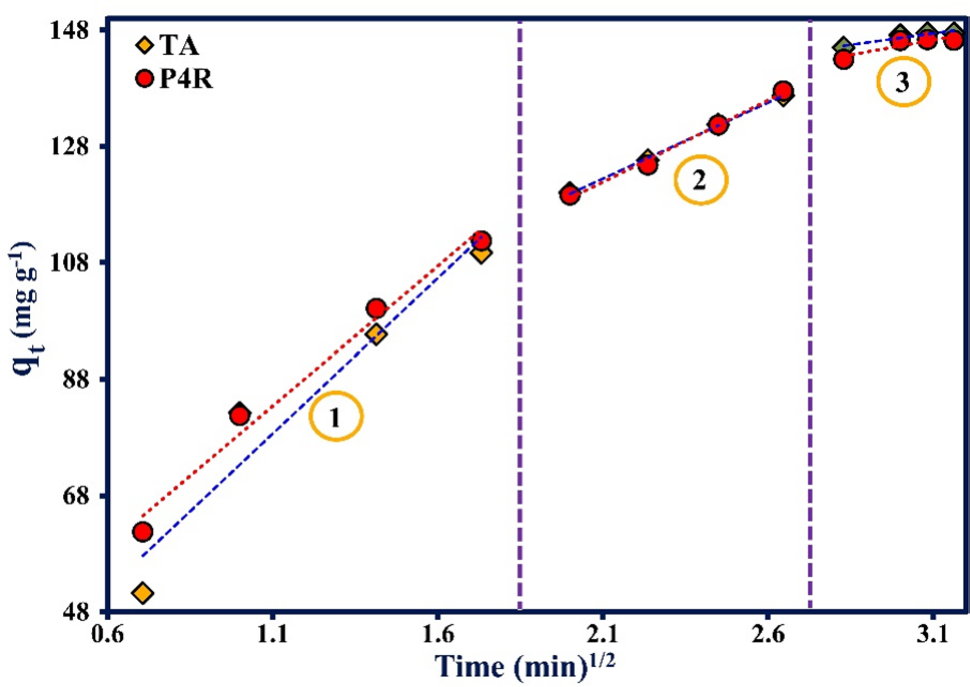
Weber-Morris intraparticle diffusion plot for the adsorptive removal of TA and P4R food dyes by ZnS/CuO-CNT.

Although the adsorption process of TA and P4R by ZnS/CuO-CNT was followed well by the Pseudo-second-order-kinetic model, however, the diffusion mechanism and the rate-controlling step in the adsorption process are not still confirmed. Therefore, the Weber-Morris model was used to study the actual rate-controlling steps in the adsorption process. The Weber-Morris fitted plot of qt against t^1\2^ is shown in Fig. 10. The result illustrates a multilinear plot in three sections, which confirmed that the adsorption of TA and P4R by ZnS/CuO-CNT was controlled by a three-stage mechanism. The first stage of adsorption had a higher slope and up to 1.8 (min)^1/2^, which was related to the boundary layer diffusion of dye molecules onto the surface of the adsorbent. Then, the slope of the straight section decreased gradually in the second stage and showed a gradual linear adsorption fit between 1.9 and 2.8 (min)^1/2^, which was related to the diffusion of dye molecules into the interior pore structure of the adsorbent. Finally, the slope of the straight section approached almost zero in the last stage of adsorption from 2.9 to 3.2 (min)^1/2^, which confirmed that the adsorption process reached the equilibrium condition. In this stage, all active site of the adsorbent was wholly saturated with dye molecules. Also, the non-zero values of Ci at all the stages showed that the adsorption process was not only intra-particle diffusion, and other interaction mechanisms including adsorption on the external surface might be acting simultaneously to it^68^.

### Thermodynamic parameters

In the adsorption process, the determination of thermodynamic parameters is important to detecting the endothermic or exothermic of the adsorption process as well as for determining its spontaneity. In thermodynamic studies of the adsorption process, it is necessary to determine three parameters, standard enthalpy (∆H°), standard Gibbs free energy (∆G°), and standard entropy (∆S°)^67^. Thermodynamic variables at different temperatures were investigated and the results are collected in Table 6. The positive values of ∆H°, indicating that the adsorption process of the dyes by ZnS/CuO-CNT was an endothermic reaction. Also, positive values of ∆S° indicate an increase in irregularity in the solid-soluble surface during the adsorption process. In other words, the positive entropy changes of the standard system indicate an increase in irregularities in ZnS/CuO-CNT in the adsorption process of P4R and TA dyes compared to the initial state before the adsorption process. Negative changes in standard Gibbs free energy showed that the adsorption process of P4R and TA dyes was spontaneous. Also, it has become more negative with increasing temperature, which indicates that the spontaneity of the reaction has increased at higher temperatures.

**Table 6:** Adsorption thermodynamic data of ZnS/CuO-CNT towards TA and P4R food dyes.

T(k)	P4R		TA	
	K_d_	ΔG°( kJ mol^-1^)	K_d_	ΔG°( kJ mol^-1^)
278.15	22.27	− 7.172	25.00	− 7.437
283.15	37.85	− 8.549	39.91	− 7.966
288.15	66.42	− 10.047	88.75	− 10.74
298.15	145.0	− 12.33	161.7	− 12.60
308.15	245.1	− 14.09	245.3	− 14.09
ΔS°(J mol^-1^ k^-1^)	215.2		205.0	
ΔH°(kJ mol^-1^)	55.66		52.89	

### Proposed adsorption mechanism

**Figure 11:**
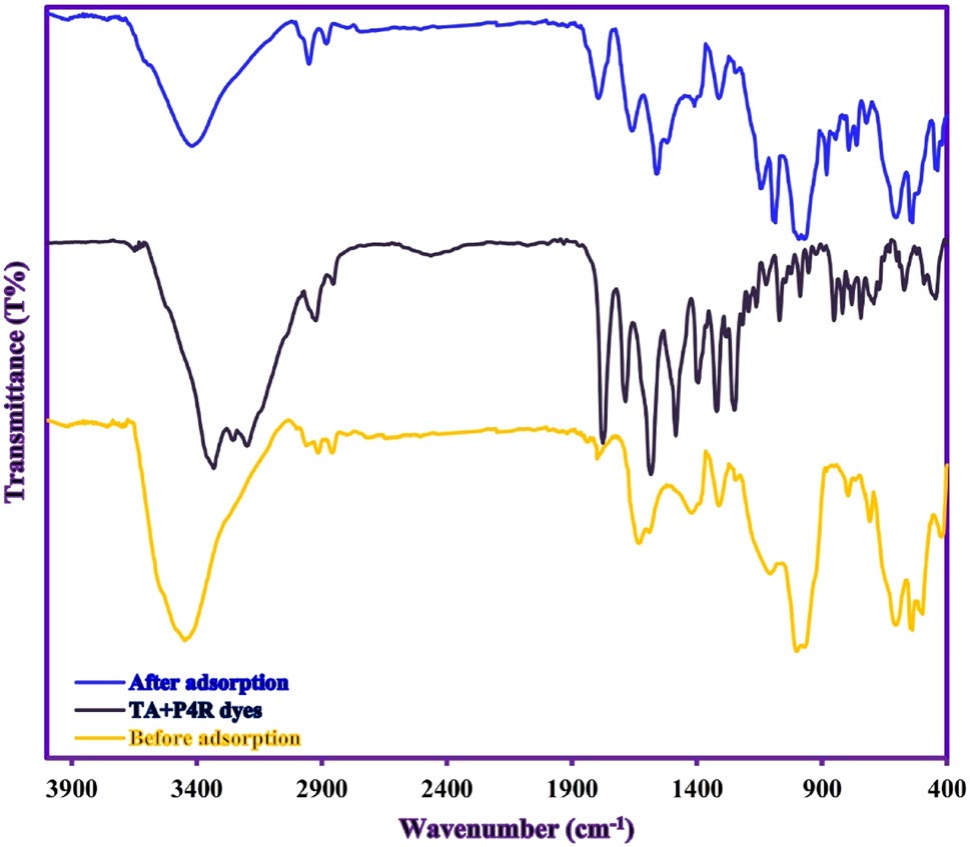
FTIR spectra of ZnS/CuO-CNT (before and after adsorption of TA + P4R dyes).

To better understand the possible adsorption mechanism, isotherm data, and adsorption kinetics were examined and showed that the adsorption of P4R and TA dyes by ZnS/CuO-CNT can occur due to both physical and chemical reactions as follows: (i) the slope (1/n) of Freundlich isotherm was less than one for both dyes and indicated the main process of the adsorption was chemisorption. (ii) The adsorption energy (E) of Dubinin-Radushkevich isotherm, indicating that the adsorption process of TA and P4R dyes exhibit chemical and physical adsorption behaviors, respectively. (iii) better agreement of the adsorption data with the Pseudo-second-order-kinetic model shows that the chemisorption is the dominant control mechanism for both dyes. Also, the FTIR spectra of ZnS/CuO-CNT before and after adsorption of TA and P4R dyes are present in Fig. 11. The result showed that the C–O, C = C, − COOH, C–H, O–H, CuO, and ZnS functional groups of ZnS/CuO-CNT shifted after the adsorption process, which could indicate the involvement of hydrogen bonding, π-π, n-π, and electrostatic interaction mechanisms in the adsorption process. On the other hand, the appearance of absorption peaks related to the TA and P4R dyes in the after adsorption FTIR spectrum of ZnS/CuO-CNT also indicates the presence of chemisorption process^69^.

### Adsorption of TA and P4R food dyes in real effluents

**Figure 12:**
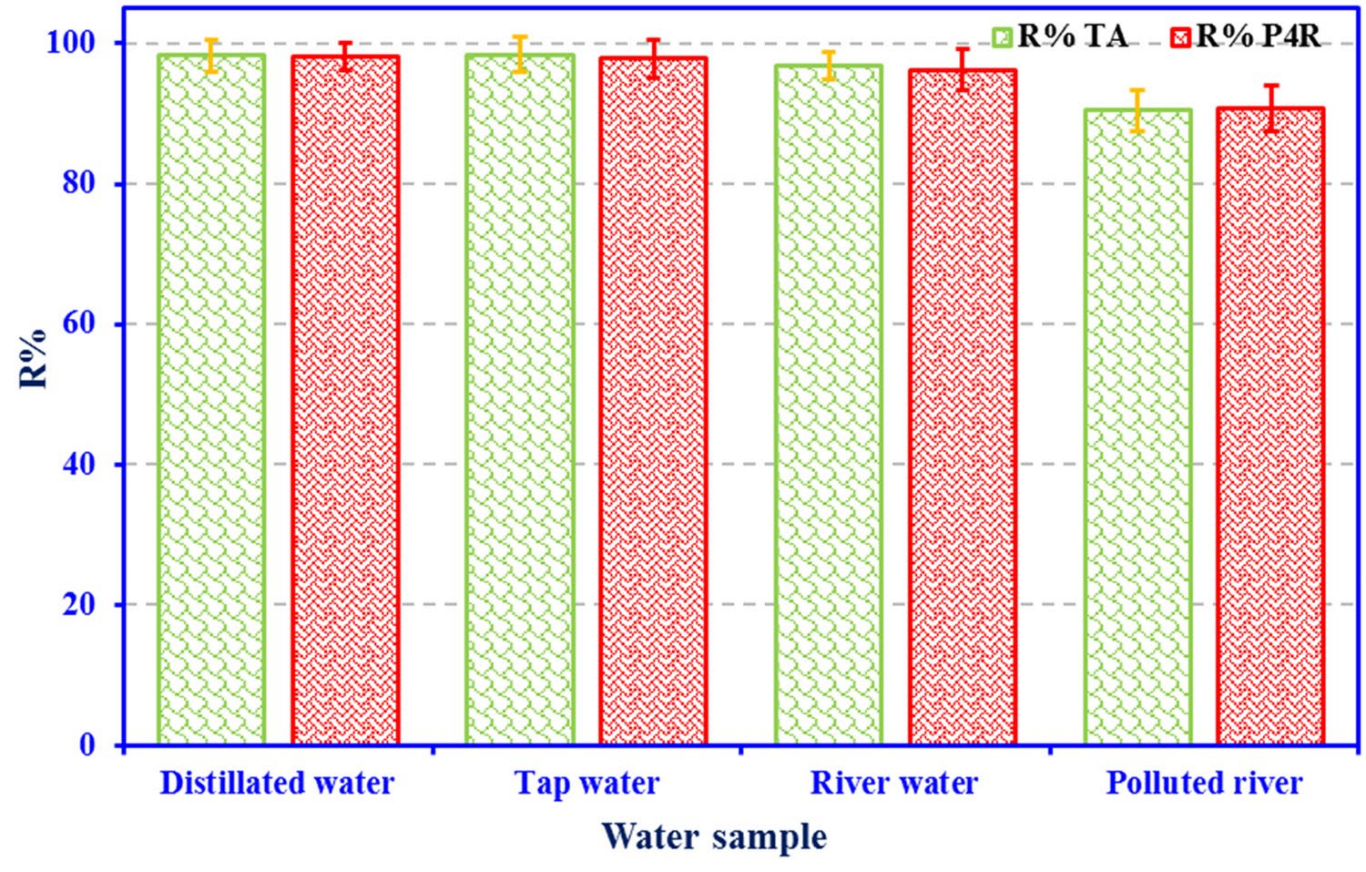
Removal efficiency of ZnS/CuO-CNT for TA + P4R dyes in various water samples.

To evaluate the adsorbent performance in real conditions, the simultaneous removal of P4R and TA dyes was investigated using ZnS/CuO-CNT adsorbent in distilled water and also real effluents including tap water, river water, and polluted water. Figure 12 shows the characteristics of the effluent following adsorption with the ZnS/CuO-CNT adsorbent. As can be observed, adsorbent has good effectiveness in removing binary combination dyes at the same time and was able to remove over 90% of dye residuals. The results demonstrated the ZnO/CuO-CNT can be very successful in treating dye effluents in real environmental conditions.

### Adsorbent regeneration and reuse experiments

**Figure 13:**
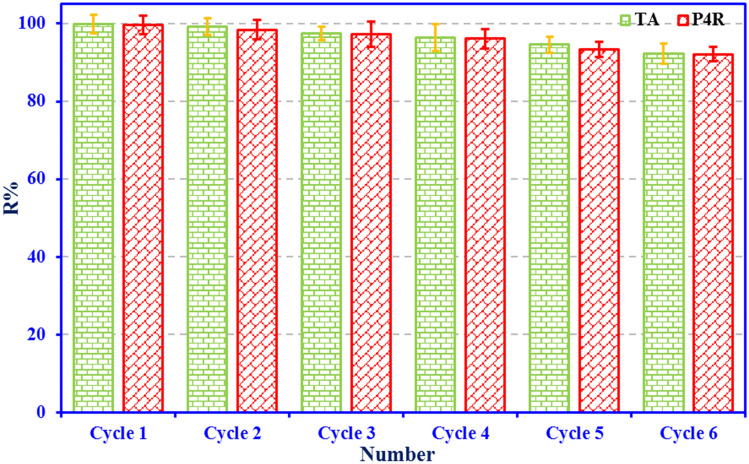
Cycling runs of TA and P4R removal by ZnS/CuO-CNT.

The regenerate ability and reused the spent adsorbent is very critical in economical applications. For this purpose, the dye-loaded adsorbent was regenerated by using the desorbing agent of NaOH (0.1 mol L ^−1^)/ethanol at ambient temperature, washed with ultrapure water, and after being dried in the oven (120 ºC for 1 h) was reused in the next cycle of adsorption experiments. Figure 13 shows the experimental results of the dyes removal efficiency by the ZnS/CuO-CNT adsorbent in each adsorption–desorption cycle. As can be seen, absorbent reuse was performed in six stages of the adsorption/desorption cycles. The results showed that the regenerated adsorbent after the five adsorption–desorption re-cycles could still remove dyes more than 90% from the aqueous solution. Further, the relatively high desorption efficiency and good regeneration of adsorbent by desorption agent indicate that the adsorptive removal of dyes by the adsorbent may have mostly occurred by electrostatic interactions^70^, which corroborates well the explanation of the initial solution pH effect on dyes removal efficiency investigated in section ‘Simultaneous effect of independent variables on dyes removal efficiency’.

### Comparison of maximum removal capacity with different adsorbents

One of the things that should be considered in the performance evaluation of an adsorbent is the study of the maximum adsorption capacity (Q_max_) of the prepared adsorbent compared to other common adsorbents. Table 7 shows the results of the comparison Q_max_ of the ZnS/CuO-CNT adsorbent with other adsorbents. As can be seen, the ZnS/CuO-CNT has a good Qmax for both P4R and TA dyes and is competitive with other reported adsorbents in terms of performance.

**Table 7:** Comparison of maximum adsorption capacity (Q_max_) of some various adsorbent for P4R and TA food dyes.

Adsorbent	Q_max_ (mg g^-1^)		References
	P4R	TA	
Amine functionalized Kit-6 mesoporous magnetite nanocomposite	87.7	–	^71^
PPy/CS/GO nanocomposite	5.40	–	^14^
Mg/Al-layered double hydroxide	45.67	–	^72^
Ultrasound-assisted treatment of chitin	2.0	–	^73^
Low-cost agricultural by-product (Saw dust)	–	4.71	^25^
papaya seeds	–	51.0	^74^
Iron oxide nanoparticles	–	59.79	^75^
Activated carbon biosorbents of Lantana Camara	–	90.90	^76^
Ni-Ag NPs/ rGO	–	26.31	^29^
Mn_0.4_Zn_0.6_Fe_2_O_4_-NPs-D-YL-ISF7	101.5	90.83	^52^
ZnS/CuO-CNT	190.1	183.5	This work

## Conclusion

In this study, the ZnS/CuO-CNT nanocomposite was synthesized by the in-situ hydrothermal synthesis, and its performance as a potential adsorbent in the simultaneous ultrasound-assisted adsorptive removal of a binary mixture of P4R and TA dyes from contaminated waters was systematically investigated. The physico-chemical characteristics of synthesized adsorbent were studied by XRD, FESEM, FTIR, BET, and ZP analysis. The independent process variables including adsorbent dosage, initial solution pH, sonication time, initial dyes concentration, and temperature were considered and statistically optimized to maximize P4R and TA dyes adsorptive removal efficiency using the CCD-RSM design. Based on ANOVA analysis, the experiment was systematically adopted with the confidence level of *p* < 0.05, and the statistically significant model was developed for dyes removal through regression analysis (R^2^ = 0.99). The optimal values were adsorbent doseage of 10 mg, pH value of 2.0, sonication time of 12 min, dyes concentration of 30.0 mg L^−1^, and solution temperature of 25 °C. The results showed that the removal efficiency increased at acidic pH and is also directly related to the sonication time, adsorbent dosage, and temperature. In contrast, increasing the concentration of dyes reduces the removal efficiency. Under the optimum conditions, the predicted R% response for TA and P4R dyes was above 99.0% with a desirability function of 1.0. Also, the experimental R% for TA and P4R were found to be 99.21 ± 2.23, and 98.45 ± 2.54, respectively. The optimum conditions were applied to study the adsorption isotherms, kinetics and thermodynamics. In addition, the adsorbent reusability results revealed that the adsorbent after the five adsorption–desorption re-cycles could still remove dyes more than 90% from the aqueous solution.
